# The Protective Role of *Magnoliae Flos* in Preventing Ovotoxicity and Managing Ovarian Function: An In Vitro and In Vivo Study

**DOI:** 10.3390/ijms25126456

**Published:** 2024-06-12

**Authors:** Mi Ra Kim, Dong-Il Kim, Sung Yun Park, Hyo Jin Kang, Sun-Dong Park, Ju-Hee Lee

**Affiliations:** 1 College of Korean Medicine, Dongguk University, Goyang 10326, Republic of Korea; okok8030@naver.com (M.R.K.); obgykdi@hanmail.net (D.-I.K.); bmepark@dongguk.ac.kr (S.Y.P.); 2 Department of Biomedical Laboratory Science, Honam University, Gwangju 62399, Republic of Korea; hyojinkang.bio@gmail.com

**Keywords:** *Magnoliae Flos*, 4-vinylcyclohexene diepoxide, ovotoxicity, ovary, premature ovarian insufficiency, diminished ovarian reserve

## Abstract

*Magnoliae Flos* (MF) is a medicinal herb widely employed in traditional medicine for relieving sinusitis, allergic rhinitis, headaches, and toothaches. Here, we investigated the potential preventive effects of MF extract (MFE) against 4-vinylcyclohexene diepoxide (VCD)-induced ovotoxicity in ovarian cells and a mouse model of premature ovarian insufficiency (POI). The cytoprotective effects of MFE were assessed using CHO-K1 or COV434 cells. In vivo, B6C3F1 female mice were intraperitoneally injected with VCD for two weeks to induce POI, while MFE was orally administered for four weeks, beginning one week before VCD administration. VCD led to a significant decline in the viabilities of CHO-K1 and COV434 cells and triggered excessive reactive oxygen species (ROS) production and apoptosis specifically in CHO-K1 cells. However, pretreatment with MFE effectively prevented VCD-induced cell death and ROS generation, while also activating the Akt signaling pathway. In vivo, MFE increased relative ovary weights, follicle numbers, and serum estradiol and anti-Müllerian hormone levels versus controls under conditions of ovary failure. Collectively, our results demonstrate that MFE has a preventive effect on VCD-induced ovotoxicity through Akt activation. These results suggest that MFE may have the potential to prevent and manage conditions such as POI and diminished ovarian reserve.

## 1. Introduction

In parallel with the expanded role of women in society, marriage and childbearing are being delayed, and female infertility is increasing [1]. Recently, decreased ovarian function, including diminished ovarian reserve and premature ovarian insufficiency (known alternatively as premature ovarian failure), has rapidly emerged as a major causative factor of infertility in women [2,3]. In the majority of cases, decreased ovarian function and premature ovarian insufficiency are commonly linked with chemotherapy, radiotherapy, autoimmune diseases, and ovariectomy [4]. According to statistics issued by the Health Insurance Review and Assessment Service in South Korea, the number of patients in their 20s and 30s with premature ovarian insufficiency has increased 2.2- and 1.8-fold over the past 12 years (2010–2022) [5,6].

Premature ovarian insufficiency (POI) is described as the cessation of ovarian function occurring before the age of 40 and, like menopause, is accompanied by symptoms such as amenorrhea, hypergonadotropic hypogonadism, and estrogen deficiency [7]. POI is typically idiopathic and may respond to treatment, but as symptoms emerge, the progression accelerates, demanding prompt intervention [8]. At its onset, POI brings forth a range of menopausal symptoms such as menstrual irregularities, hot flashes, and insomnia, as well as depression, emotional instability, reduced appetite, and vaginal dryness. POI also increases the risks of osteoporosis, cardiovascular disease, and infertility [9].

Women with the diagnosis of POI are typically recommended to pursue prolonged conventional hormone replacement therapy until the average age of normal natural menopause. While this can address the lack of estrogen and alleviate symptoms associated with menopause, it fails to restore ovarian function [10,11]. POI patients with intact uteruses are generally prescribed estrogen with progesterone to prevent the risk of endometrial hyperplasia and estrogen alone after hysterectomy [12]. However, long-term estrogen plus progesterone treatment has been linked to elevated risks of cardiovascular disease, stroke, and breast cancer [13]; therefore, there is increasing demand for alternative therapies.

In traditional East Asian medicine, herbal medicines have been mainly prescribed for the treatment of POI, along with auxiliary treatments such as acupuncture, pharmacopuncture, and moxibustion [14]. Several studies have suggested that complementary and alternative treatments, including herbal medicines, may improve POI symptoms [15,16,17,18,19,20], but evidence of their efficacy and safety is insufficient. Therefore, novel treatment strategies incorporating complementary and alternative therapies need to be investigated to optimize the treatment of POI. Recently, interest in natural materials has increased, and developments based on their use are being pursued in various fields, such as pharmaceuticals and functional foods.

**Figure 1:**
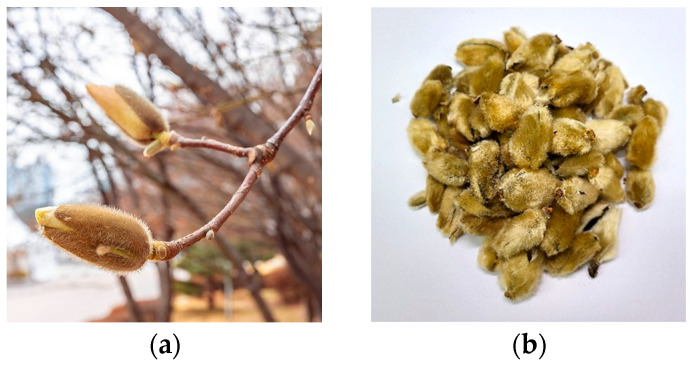
Pictures of *Magnoliae Flos* (**a**) and its dried flower buds (**b**).

*Magnoliae Flos* (MF, also known as Shinyi in Korean) is the dried flower buds of *Magnolia biondii* Pampanini, *Magnolia denudata* Desrousseaux, *Magnolia kobus* De Candolle, and *Magnolia sprengeri* Pampanini, which belong to the Magnoliaceae family and grow wild in East Asia, including Korea, China, and Japan (Figure 1). In traditional medicine, MF has been used to treat allergic rhinitis, sinusitis, nasal congestion, headaches, and toothache and is prescribed as Shinyicheongpye-tang (辛夷淸肺湯) or Shinyisan (辛夷散) [21,22]. Several studies have reported that MF has anti-allergic, anti-inflammatory, antibacterial, antioxidant, and neuroprotective activities and inhibitory effects on ovariectomy-induced osteoporosis [23,24,25,26,27]. Its active compounds include neolignans, magnolin, magnolol, eudesmin, vanillic acid, and fargesin, and these are reported to have anti-inflammatory, antibacterial, anti-allergic, and anti-obesity effects [28,29,30,31,32,33]. However, the effects of MF on the female reproductive system have not been addressed. In a previous study, we devised an in vitro screening method to identify potential new drug candidates aimed at preventing and treating POI [15]. 4-vinylcyclohexene diepoxide (VCD), a hazardous occupational and environmental chemical, has been considered as an ovotoxic substance for its capacity to selectively destroy ovarian primordial and primary follicles and can trigger POI [34]. Thus, the ovoprotective effects of MF extract (MFE) and the responsible mechanism were investigated in both a cell-based VCD ovotoxicity model and a VCD-induced POI mouse model.

## 2. Results

### 2.1. Effects of MFE on the Viability of CHO-K1 and COV434 Cells

**Figure 2:**
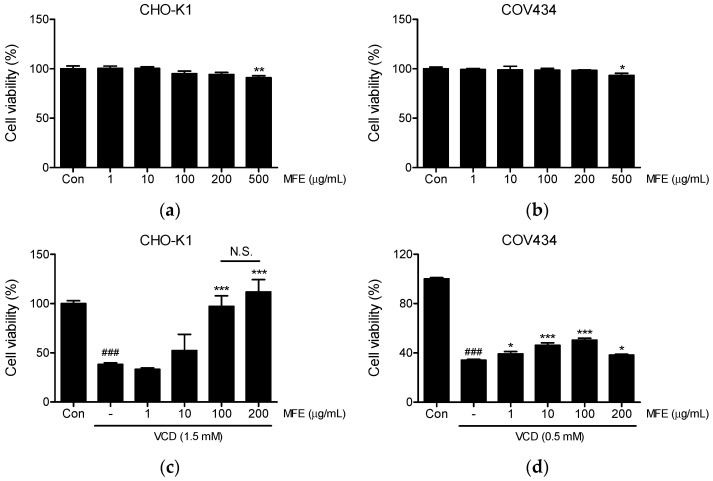
Effects of *Magnoliae Flos* extract (MFE) on the viability of CHO-K1 and COV434 cells. (**a**,**b**) Cytotoxic effects of MFE. CHO-K1 (**a**) and COV434 (**b**) cells were treated with various concentrations of MFE (1, 10, 100, 200, or 500 µg/mL) for 24 h. (**c**,**d**) Effects of MFE on 4-vinylcyclohexene diepoxide (VCD)-induced ovotoxicity. CHO-K1 (**c**) and COV434 (**d**) cells were initially pretreated with different concentrations (1–200 μg/mL) of MFE for 1 h, followed by exposure to either 1.5 mM or 0.5 mM VCD for 24 h, respectively. Subsequently, the MTT assay was conducted to measure cell viability, and the resulting values were expressed as percentages in comparison to vehicle-treated controls (significant vs. vehicle-treated controls, ^###^ *p* < 0.001; significant vs. VCD-treated cells, * *p* < 0.05, ** *p* < 0.01, *** *p* < 0.001).

To assess the cytotoxic effects of MFE, we initially conducted an MTT assay to evaluate its effects on the viability of CHO-K1 and COV434 cells after treatment with MFE. MFE treated at 1 to 200 µg/mL for 24 h had no toxic effect on CHO-K1 (Figure 2a) or COV434 (Figure 2b) cells, but at 500 µg/mL, viabilities were slightly reduced to 90% (*p* < 0.01) and 93% (*p* < 0.05), respectively. Based on these results, MFE was used at concentrations ≤ 200 μM in subsequent experiments on CHO-K1 and COV434 cells.

Next, we evaluated whether MFE has an ovoprotective effect using a cell-based screening system we previously established [15]. To investigate the protection afforded by MFE against VCD, CHO-K1 and COV434 cells were pretreated with MFE at concentrations ranging from 1 to 200 µg/mL for 1 h. Subsequently, they were exposed to either 1.5 mM or 0.5 mM VCD for 24 h, respectively. As illustrated in Figure 2c, pretreatment with MFE at 100 and 200 µg/mL significantly protected CHO-K1 cells from the toxic effects induced by VCD, with no significant difference between the two concentrations. Furthermore, it notably protected COV434 cells against VCD-induced ovotoxicity at all tested concentrations and dose-dependently increased cell viability within the range of 1 to 100 µg/mL (Figure 2d). At 200 µg/mL, its protective effect was comparable to that at 1 µg/mL (Figure 2d).

### 2.2. Inhibitory Effects of MFE on VCD-Induced Reactive Oxygen Species (ROS) Generation

**Figure 3:**
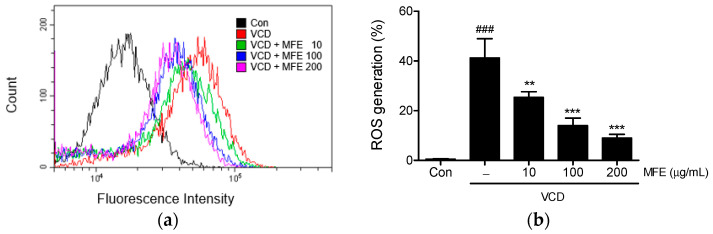
Effects of MFE on VCD-induced reactive oxygen species (ROS) generation in CHO-K1 cells. CHO-K1 cells were pretreated with different concentrations (10–200 μg/mL) of MFE for 1 h, followed by exposure to 1.5 mM VCD for 24 h. The assessment of intracellular ROS levels was conducted using flow cytometry following the staining of cells with DCFH_2_-DA dye. A representative figure (**a**) and the accompanying histogram (**b**) demonstrate the inhibitory effects of MFE on VCD-induced ROS generation. Significant vs. vehicle-treated controls, ^###^ *p* < 0.001; significant vs. VCD-treated cells, ** *p* < 0.01, *** *p* < 0.001.

To examine the cellular antioxidant capacity of MFE, intracellular ROS levels were measured in VCD-treated CHO-K1 cells. Exposure to VCD resulted in a significant increase in intracellular ROS levels (41.2 ± 7.79%), which were dose-dependently suppressed by MFE pretreatment (Figure 3). In particular, MFE at 100 and 200 µg/mL effectively suppressed ROS generation to a comparable extent (14.01 ± 3.00% vs. 8.99 ± 1.50%, respectively), with no statistically significant difference observed.

### 2.3. Inhibitory Effects of MFE on VCD-Induced Apoptosis

**Figure 4:**
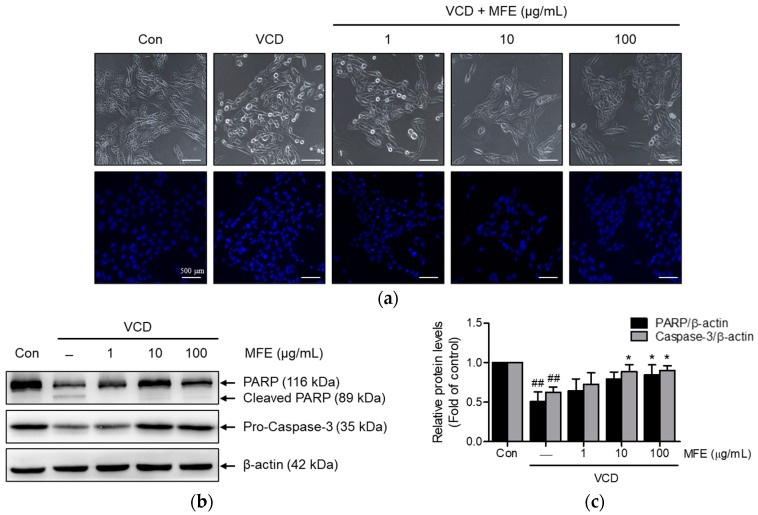
Effects of MFE on VCD-induced apoptosis in CHO-K1 cells. (**a**) Effects of MFE on morphological alterations of whole cells and the nuclei of CHO-K1 cells treated with VCD. CHO-K1 cells were pretreated with different concentrations (1–100 μg/mL) of MFE for 1 h, followed by exposure to 1.5 mM VCD for 24 h. Scale bar 500 µm. (**b**) Effects of MFE on apoptosis-related proteins in CHO-K1 cells treated with VCD. Cells were treated as described in (**a**), and PARP and caspase-3 expressions were determined by Western blot. (**c**) Quantification of Western blot bands. The intensity of each band was measured using densitometry with ImageJ 1.52a software and normalized to β-actin. Results are presented as the ratio of the experimental group to the control group, with the control group value set to 1. Significant vs. vehicle-treated controls, ^##^ *p* < 0.01; significant vs. VCD-treated cells, * *p* < 0.05.

A fluorescence microscope was used to investigate the effects of MFE on VCD-induced apoptosis-associated morphological changes in 4,6-Diamidino-2-phenylindole (DAPI)-stained CHO-K1 cells. Following VCD treatment, most cells showed apoptotic characteristics, such as condensed or fragmented nuclei. However, MFE pretreatment dose-dependently mitigated these features (Figure 4a). Additionally, VCD treatment led to increased poly (ADP-ribose) polymerase (PARP) cleavage and decreased pro-caspase-3 levels. Notably, MFE pretreatment fully prevented these changes at concentrations of 10 and 100 μg/mL (Figure 4b,c).

### 2.4. Effects of MFE on Akt Signaling Pathway in CHO-K1 Cells

**Figure 5:**
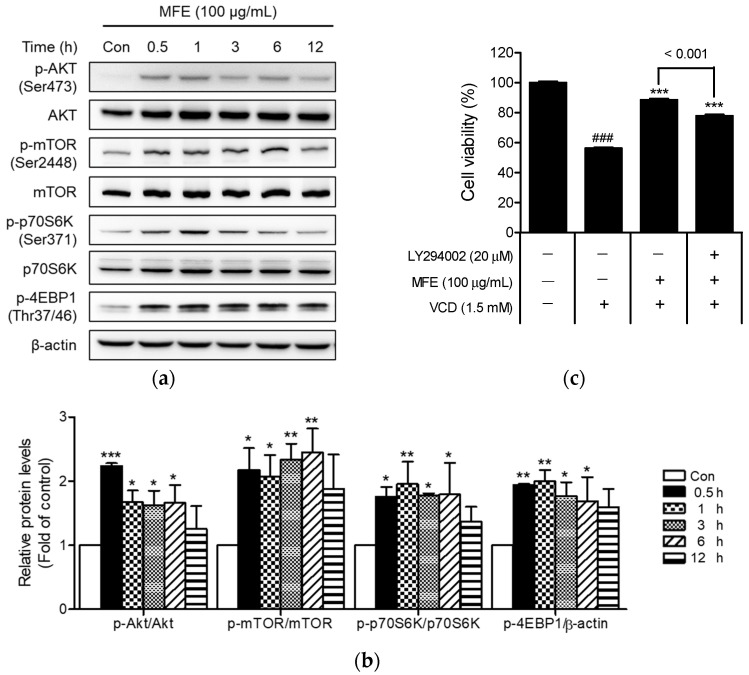
Effects of MFE on the Akt signaling pathway in CHO-K1 cells. (**a**) Representative images of Western blotting bands for Akt signaling pathway-related proteins. CHO-K1 cells were treated with 100 µg/mL MFE for the specified durations, and extracted proteins were immunoblotted using antibodies related to the Akt signaling pathway. (**b**) Quantification of Western blot bands. The intensity of each band was measured using densitometry with ImageJ 1.52a software and expressed as the ratios of p-Akt/Akt, p-mTOR/mTOR, p-p70S6K/p70S6K, and p-4EBP1/β-actin. Results are presented as the ratio of the experimental group to the control group, with the control group value normalized to 1. Significant vs. vehicle-treated controls, *** *p* < 0.001, ** *p* < 0.01, * *p* < 0.05. (**c**) Attenuation of the protective effect of MFE by LY294002. After pretreating the cells with LY294002 (20 µM) for 1 h, they were then treated with 100 µg/mL MFE for 1 h, followed by exposure to 1.5 mM of VCD for 24 h. Subsequently, cell viability was measured. Significant vs. vehicle-treated controls, ^###^ *p* < 0.001; significant vs. VCD-treated cells, *** *p* < 0.001.

The effects of MFE on the Akt signaling pathway were examined by Western blotting to elucidate the mechanism underlying its cytoprotective effects. At 100 µg/mL, MFE markedly triggered the phosphorylation of Akt and its downstream target molecules, viz. mTOR, p70S6K, and 4EBP1, after 30 min of treatment (Figure 5a). These phosphorylations peaked at 0.5 or 1 h and gradually subsided over 12 h (Figure 5b).

Subsequently, the role of Akt activation in MFE’s cytoprotective effect was explored by treating CHO-K1 cells with LY294002 (20 µM, a PI3K/Akt inhibitor). As illustrated in Figure 5c, the results of the cell viability measurement show that the protective effect of MFE against VCD-induced toxicity was partially attenuated by pretreatment with LY294002. These findings suggest that MFE’s protective mechanism is, at least partially, mediated by Akt activation.

### 2.5. Effects of MFE Administration on VCD-Induced Ovarian Failure in Mice

**Figure 6:**
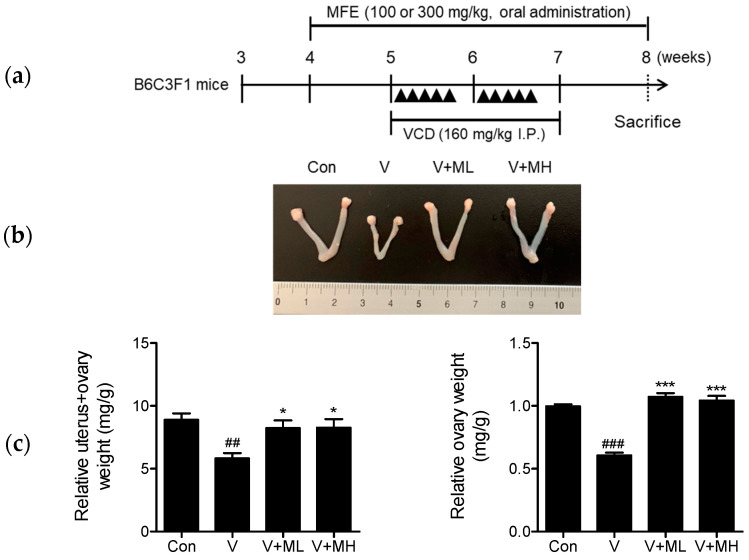
Effects of MFE administration on B6C3F1 mice with VCD-induced ovarian failure. (**a**) Procedures of the animal experiment. Control mice received an i.p. injection of sesame oil, while VCD and VCD + MFE mice received an i.p. injection of VCD (160 mg/kg) five times a week for two weeks. Additionally, starting one week before the first VCD injection, mice were orally administered either 100 or 300 mg/kg of MFE five times a week for four weeks. Three weeks after the first VCD injection, mice were sacrificed, and their ovaries and uteri were excised. (**b**) Representative photographs of ovaries and uteri from each study group. (**c**) Weight measurements. After sacrifice, uteri with one ovary attached and single ovaries were weighed. These weights were then expressed as relative values by dividing them by body weights. Results are presented as means ± SEMs. Groups: the control group (Con, n = 6), the VCD group (V, n = 6), the VCD plus MFE 100 mg/kg group (V + ML, n = 6), and the VCD plus MFE 300 mg/kg group (V + MH, n = 6). Significant vs. the control group, ^##^ *p* < 0.01, ^###^ *p* < 0.001; significant vs. the VCD group, * *p* < 0.05, *** *p* < 0.001.

In order to determine the ability of MFE to protect against ovarian failure, female B6C3F1 mice were orally administered 100 or 300 mg/kg of MFE 5 times a week for 4 weeks from 1 week before the first VCD injection (Figure 6a). Representative morphologies of excised uteri and ovaries obtained the day after the final doses are shown in Figure 6b. In addition, weights of uteri with one of the ovaries attached (uterus + ovary) and of the other ovary were recorded, and then, the relative weights were calculated using their weights divided by body weight. The relative weights of the uterus and ovary, as well as those of the ovary, were notably lower in the VCD group compared to the control group mice (Con vs. VCD, uterus + ovary: *p* < 0.01, ovary: *p* < 0.001). However, administration with MFE at 100 or 300 mg/kg prevented these reductions caused by VCD exposure (VCD vs. VCD + ML (MFE low dose) or VCD + MH (MFE high dose), uterus + ovary: *p* < 0.05, ovary: *p* < 0.001) (Figure 6c).

**Figure 7:**
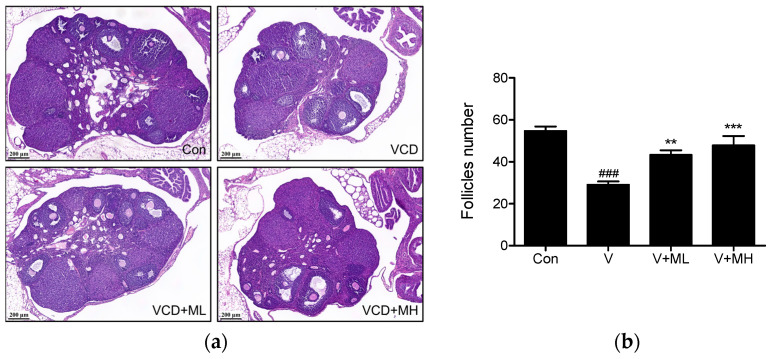
Effects of MFE administration on histological changes in ovaries in the mouse model of VCD-induced ovarian failure. (**a**) Representative histological images of ovaries stained with H&E. Scale bar 200 µm. (**b**) The number of follicles was counted and compared in the four groups. Results are presented as means ± SEMs. Groups: the control group (Con, n = 6), the VCD group (V, n = 6), the VCD plus MFE 100 mg/kg group (V + ML, n = 6), and the VCD plus MFE 300 mg/kg group (V + MH, n = 6). Significant vs. the control group, ^###^ *p* < 0.001; significant vs. the VCD group, ** *p* < 0.01, *** *p* < 0.001.

Ovaries were stained with hematoxylin and eosin (H&E) to investigate whether MFE affected ovarian function in our VCD-induced ovarian failure model. Representative histological images of ovaries in each group are shown in Figure 7a. Follicle numbers in the VCD group were notably reduced compared to the control group (*p* < 0.001). In contrast, in the VCD + ML and VCD + MH groups, they were significantly higher compared to the VCD group (*p* < 0.01 and *p* < 0.001, respectively), as illustrated in Figure 7b.

### 2.6. Effects of MFE Administration on Serum Hormone Levels and Hepatoxicity in the VCD-Induced Ovarian Failure Model

**Figure 8:**
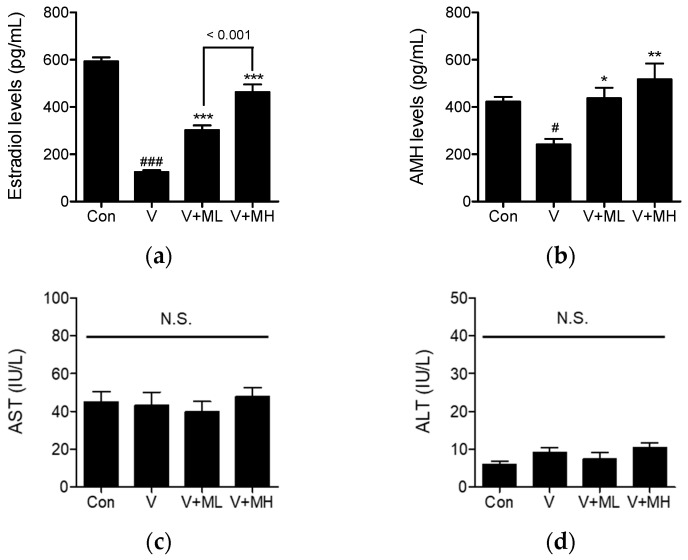
Effects of MFE administration on serum E_2_, AMH, AST, and ALT levels in a murine model of VCD-induced ovarian failure. Serum levels of E_2_ (**a**) and AMH (**b**) were assayed using ELISA kits. Changes in the serum levels of AST (**c**) and ALT (**d**) serve as indicators of liver damage. Groups: the control group (Con, n = 6), the VCD group (V, n = 6), the VCD plus MFE 100 mg/kg group (V + ML, n = 6), and the VCD plus MFE 300 mg/kg group (V + MH, n = 6). Significant vs. the control group, ^#^ *p* < 0.05, ^###^ *p* < 0.001; significant vs. the VCD group, * *p* < 0.05, ** *p* < 0.01, *** *p* < 0.001; N.S., not significant.

Serum levels of estradiol (E_2_) and anti-Müllerian hormone (AMH) were assayed in all groups. The serum E_2_ concentrations in the control, VCD, VCD + ML, and VCD + MH groups were 592.9 ± 42.1, 125.1 ± 17.5, 302.0 ± 50.4, and 462.8 ± 79.6 pg/mL, respectively (Figure 8a). The E_2_ level in the VCD group significantly decreased compared to the control group (*p* < 0.001). However, MFE administration effectively prevented this reduction in a dose-dependent manner. Specifically, both the VCD + ML and VCD + MH groups exhibited significantly higher E_2_ levels compared to the VCD group (*p* < 0.001 for both), with a significant difference observed between the VCD + ML and VCD + MH groups (*p* < 0.001). Furthermore, the serum AMH level was markedly lower in the VCD group compared to the control group (*p* < 0.05). However, it significantly increased in both the VCD + ML and VCD + MH groups compared to the VCD group (*p* < 0.05, *p* < 0.01, respectively) (Figure 8b). Specifically, low- and high-dose MFE treatments increased AMH levels by 80.9% and 114.1%, respectively, compared to the VCD group.

Next, to assess whether MFE administration for 4 weeks had a hepatotoxic effect in the VCD-induced ovarian failure model, we measured aspartate aminotransferase (AST) and alanine aminotransferase (ALT) enzyme levels (indicators of liver damage) in serum. As shown in Figure 8c,d, no significant intergroup differences were observed in AST or ALT levels. These results indicate that MFE treatment at 100 and 300 mg/kg for 4 weeks does not induce liver toxicity in B6C3F1 mice with VCD-induced ovarian failure.

## 3. Discussion

Premature ovarian insufficiency (POI) is a disorder of the female reproductive system caused by various factors, including genetic, iatrogenic, metabolic, infectious, environmental, and idiopathic factors, and after symptom onset, the progression of the disease becomes increasingly uncontrollable [9]. Although POI is not life-threatening, it significantly impacts the quality of life for women of childbearing age [16,35]. Therefore, it is crucial to reduce risk factors for POI and improve ovarian function through early diagnosis and treatment.

Ovotoxicity refers to the toxic effects on ovarian tissue and germ cells caused by various harmful factors. Anticancer therapeutics such as cyclophosphamide and cisplatin, as well as polycyclic aromatic hydrocarbons like 7, 12-dimethylbenz[a]anthracene and benzo(a)pyrene, are considered representative ovotoxic factors. These substances can trigger ovarian aging, cause premature ovarian failure, and reduce fertility [36,37,38,39]. 

Among the ovotoxic substances, VCD (4-vinylcyclohexene diepoxide) is known for its hazardous, carcinogenic properties and its selective destruction of ovarian primordial and primary follicles, leading to POI [34]. Furthermore, VCD has been reported to damage small follicles, precipitate menopause, and cause ovarian failure [40]. In the field of menopause and aging research, a rodent model of VCD-induced transitional menopause is attracting attention because this model more closely resembles the natural menopause transition compared to ovariectomy-based models [41]. In our present study, we found that MFE protected ovarian cells and tissues against VCD-induced ovotoxicity by upregulating the PI3K/Akt signaling pathway.

MF has been traditionally used to alleviate symptoms of nasal congestion, rhinitis, sinusitis, and sinus headaches, and its pharmacological effects have been well researched. However, little information is available regarding its effects on the female reproductive system. The only study related to women’s health was performed by Jun et al., who found that MF ameliorated osteoporosis induced by ovariectomy in mice [27]. Building upon this finding, we considered that MF might protect against premature ovarian failure or menopausal syndrome. In a previous study, we established a VCD-induced cytotoxic model to identify agents that protect ovaries from ovotoxic agents [15]. As we previously reported for the water extract of *Evodiae Fructus* [15], MFE protected CHO-K1 cells (an epithelial cell line originating from the ovary of the Chinese hamster) and COV434 cells (a human granulosa cell line) from VCD-induced cytotoxicity, while also attenuating VCD-induced apoptosis and excess ROS generation in CHO-K1 cells.

The Akt signaling pathway, crucial for cell survival, is known to provide signals to resist apoptotic stimuli [42]. In addition, the KIT/KITLG (kit ligand) signaling pathway attenuates ovotoxicity caused by VCD on primordial and primary follicles [34]. Furthermore, the binding of KITLG to KIT activates the PI3K/Akt signaling pathway, which plays a role in primordial follicle recruitment and activation in mammalian oocyte growth and follicular development [43,44]. Therefore, we hypothesized that drugs capable of activating the Akt signaling pathway might protect ovaries against VCD-induced ovotoxicity. As expected, upon pretreatment with LY294002 (a chemical inhibitor of Akt), the efficacy of MFE in suppressing VCD-induced reductions in cell viability was observed to be attenuated, revealing that the cytoprotective effect of MFE is, to some extent, dependent on Akt activation.

In rodents, VCD directly targets and depletes primordial and small primary ovarian follicle pools but has minimal or no effect on other organs such as the liver, spleen, kidneys, and adrenal glands [45,46]. Several studies have reported reduced ovarian and uterine weights in rodents following VCD treatment [46,47]. Specifically, Sahambi and colleagues suggested, based on the observation that AMH levels in mice exposed to VCD were significantly reduced or undetectable, that serum AMH levels might serve as an indirect marker of the primordial follicle pool [46]. Consistent with these findings, our in vivo study revealed that VCD significantly reduced relative uterus + ovary and ovary weights, follicle numbers, and serum levels of AMH and E_2_ without inducing hepatotoxicity, with MFE administration inhibiting these reductions. E_2_ (estradiol) is primarily synthesized from testosterone through the enzymatic action of aromatase in ovarian granulosa cells [48,49]. After measuring serum estradiol levels in normal mice orally administered MFE alone (100 or 300 mg/kg) without VCD, no statistically significant difference was observed compared to the control group. Therefore, it is assumed that MFE does not affect aromatase activity. However, further research is needed to provide supporting evidence.

MFE contains several key active compounds, including vanillic acid, magnolin, eudesmin, magnolol, fargesin, and tiliroside [31]. Vanillic acid (4-hydroxy-3-methoxybenzoic acid) is a phenolic acid commonly found in plant extracts, renowned for its diverse array of beneficial properties such as antioxidant, anti-inflammatory, anticancer, neuroprotective, hepatoprotective, and cardioprotective activities [50]. Recently, Mentese and colleagues reported that vanillic acid exhibited ovoprotective effects in a cisplatin-induced rat model of ovotoxicity by activating Nrf2 [37]. Furthermore, studies have shown that the renal protective effects of magnolin in rat kidney injury models are attributed to its antioxidative, anti-inflammatory, and antiapoptotic properties [51,52], while eudesmin (a tetrahydrofurofuranoid lignan) has been found to possess neuroprotective properties in an amyloid-β-induced model of Alzheimer’s disease [53]. These findings lead us to speculate that the ovoprotective effects of MFE may be attributed to the cytoprotective activities of vanillic acid, magnolin, and eudesmin.

Verification of the safety and effectiveness of herbal medicines and medicinal herbs has become a contentious issue in many countries. A recent safety evaluation of MFE, including its side effects and toxicity, reported that the NOAEL (no observed adverse effect level) of MFE was determined to be over 3000 mg/kg for a 4-week repeated oral dose in male and female Sprague Dawley rats [54]. Furthermore, reports of harmful effects of MF in humans are rare. However, a report from China mentioned two individuals who exhibited edema and red skin wheals after the oral administration of MF decoctions [21]. In the present study, MFE treatment at doses of 100 and 300 mg/kg for 4 weeks did not cause significant changes in serum AST and ALT levels, which are indicators of hepatotoxicity.

We acknowledge that one limitation of our study is the small sample size, with only six mice per group. This limited sample size may have implications for the generalizability of our findings. Despite this limitation, our study provides valuable insights into the potential preventive effects of MFE against ovotoxicity and its role in maintaining ovarian function. The observed effects on cellular and animal models warrant further investigation in larger-scale studies and clinical trials to elucidate the mechanisms underlying MFE’s actions and its potential application in human reproductive health.

In summary, MFE can protect ovarian cells from VCD-induced cell-based ovotoxicity by activating the Akt signaling pathway. Additionally, in POI mice, MFE demonstrated the ability to reduce VCD-induced effects, including decreases in relative uterine and ovary weights, follicle numbers, as well as serum levels of E_2_ and AMH without causing hepatoxic effects. These findings collectively indicate that MFE has the potential to mitigate ovotoxicity and ovarian damage induced by VCD both in vitro and in vivo by activating Akt. Consequently, MFE may be considered as a potential treatment for female premature ovarian insufficiency and diminished ovarian reserve.

## 4. Materials and Methods

### 4.1. Preparation of Magnoliae Flos Macerated Water Extract

*Magnoliae Flos* (MF) was purchased from Humanherb (Daegu, Republic of Korea). To prepare the MF extract (MFE), dried flower buds (100 g) were ground and then soaked in water (800 mL) at room temperature for 1 week with frequent agitation. Following two filtrations with filter paper (8 μm pore size), the extract underwent concentration on a rotary evaporator (EYELA, Tokyo, Japan) under reduced pressure. The yield of dry extract after lyophilization in a freeze-dryer (EYELA) was 11% (*w*/*w*).

### 4.2. HPLC Analysis of MFE

**Figure 9:**
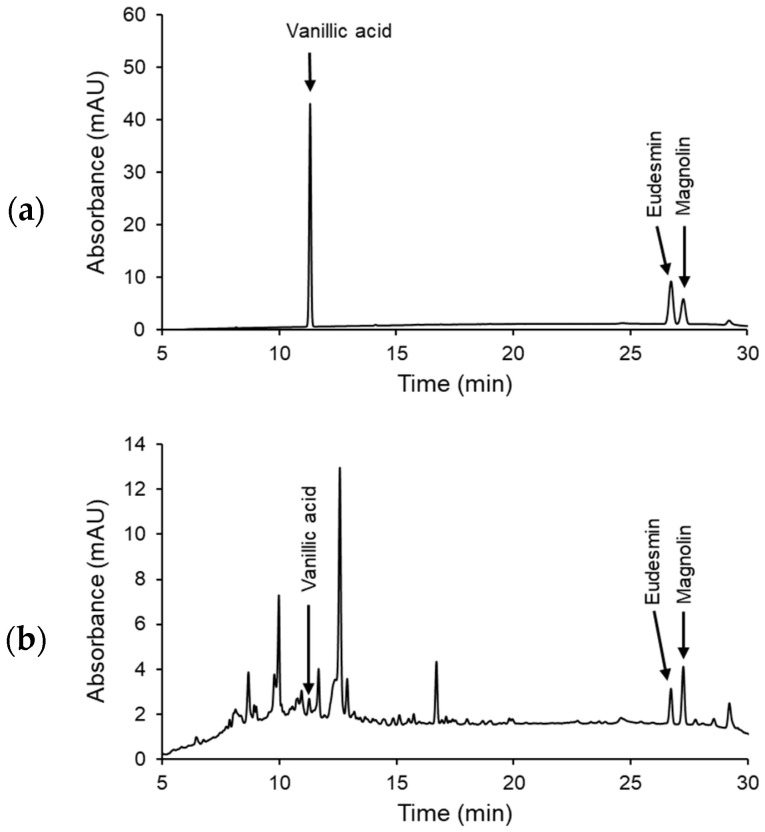
Representative HPLC chromatogram of MFE. (**a**) Chromatograms of standard compounds, vanillic acid, eudesmin, and magnolin as markers for quality control. (**b**) Chromatograms of MFE. The retention times of vanillic acid, eudesmin, and magnolin were 11.270, 26.727, and 27.253 min, respectively.

A qualitative analysis of MFE was conducted employing three marker compounds—vanillic acid (Sigma-Aldrich, St. Louis, MO, USA), eudesmin, and magnolin (Chem-Faces, Wuhan, China)—using the Dionex Ultimate™ 3000 HPLC system (Thermo Fisher Scientific, Waltham, MA, USA) as detailed in a previous study [55]. The separation of components was achieved on an INNO C-18 column (5 μm, 4.6 mm × 250 mm, Young Jin Biochrom Co., Ltd., Seongnam, Republic of Korea) maintained at a constant 30 °C. A step-gradient program was executed using a mobile phase composed of 0.1% trifluoroacetic acid (A) and acetonitrile (B): starting at 10% B for 0–25 min, transitioning to 60% B for 25–30 min, reaching 100% B for 30–35 min, and finally returning to 10% B for 36–40 min. Then, 10 µL samples were injected at a flow rate of 0.8 mL/min, with compound detection at 280 nm. Data acquisition and processing were performed using Chromeleon 7.2 chromatography data system software. A chromatogram displaying the chemical profile of MFE is presented in Figure 9. A comparison of the chromatogram of MFE with a standard chromatogram identified peaks with retention times of 11.270, 26.727, and 27.253 min, corresponding to vanillic acid, eudesmin, and magnolin, respectively.

### 4.3. Cell Culture

CHO-K1 (a Chinese hamster ovary-derived epithelial-like cell line) and COV434 (a human-derived immortalized granulosa cell line) cells were purchased from the Korean Cell Line Bank (Seoul, Republic of Korea) and Sigma-Aldrich (St. Louis, MO, USA), respectively. CHO-K1 cells were grown in DMEM (WELGENE, Gyeongsan, Republic of Korea) high glucose, with the supplementation of 10% fetal bovine serum (WELGENE) and 100 U/mL penicillin/100 μg/mL streptomycin (Thermo Fisher Scientific) in a humidified CO_2_ incubator (5% CO_2_/95% air; Thermo Fisher Scientific) at 37 °C. COV434 cells were also maintained in the same medium, which included L-glutamine (2 mM).

### 4.4. Cell Viability Assay

To investigate the cytotoxic effect of MFE, CHO-K1 and COV434 cells were seeded at a density of 5–6 × 10^3^ cells per well (100 μL) in 96-well plates, cultured for 18 h, and subsequently treated with varying concentrations of MFE (1–500 μg/mL) for an additional 24 h. To investigate the cytoprotective effect of MFE, CHO-K1 and COV434 cells were pretreated with MFE at concentrations ranging from 1 to 200 μg/mL for 1 h. Subsequently, 4-vinylcyclohexene diepoxide (VCD; Sigma-Aldrich) was added to 1.5 mM or 0.5 mM, respectively, and cells were further incubated for 24 h. Viable cells were subjected to staining with an MTT (3-(4,5-dimethylthiazol-2-yl)-2,5-phenyltetrazolium bromide) (Sigma-Aldrich) solution for 2 h. The formazan crystals produced were fully dissolved by the addition of dimethyl sulfoxide (100 μL/well), followed by determining the absorbance values at 540 nm with a microplate reader (Tecan, Research Triangle Park, NC, USA).

### 4.5. Measurement of ROS

The evaluation of intracellular ROS levels was performed through a DCFH_2_-DA (2′,7′-dichlorofluorescein diacetate) assay. CHO-K1 cells were plated at a density of 1.5 × 10^5^ cells per well on a 6-well plate and pretreated with 10–200 μg/mL of MFE for 1 h, and then, 1.5 mM of VCD (Sigma-Aldrich) was added, and cells were further incubated for 24 h. After discarding the medium, each well received a solution containing 1 μM of DCFH_2_-DA (Sigma-Aldrich) in phosphate-buffered saline (PBS), undergoing incubation for 30 min at 37 °C. After collecting the cells, fluorescence intensities were measured with a CytoFLEX flow cytometer (Beckman Coulter Inc., Brea, CA, USA), and data were processed using CytExpert software (version 2.2.0.97, Beckman Coulter Inc.).

### 4.6. DAPI Nuclear Staining

CHO-K1 cells were plated on 18 mm diameter cover glasses in 12-well plates, and 24 h later, they were pretreated with 1–100 μg/mL of MFE for 1 h. VCD (1.5 mM, Sigma-Aldrich) was then added. After incubation for 24 h, the cells were fixed by immersion in ice-cold methanol for 5 min, rinsed with PBS, and the cover glasses were affixed onto glass slides using a mounting medium containing DAPI (Vector Laboratories, Burlingame, CA, USA). Subsequently, the nuclei were examined under a fluorescence microscope (Nikon, Tokyo, Japan), and image capturing ensued.

### 4.7. Western Blot Analysis

For Western blotting, whole-cell lysates of MFE-treated CHO-K1 cells were prepared using RIPA lysis buffer (Thermo Fisher Scientific). After quantifying the protein concentrations in lysates with the Pierce™ BCA protein assay kit (Thermo Fisher Scientific), protein samples were prepared, loaded onto 8~15% SDS-PAGE, separated by electrophoresis, and then transferred to PVDF membranes (GE Healthcare Life Sciences, Freiburg, Germany). After blocking with EveryBlot blocking buffer (Bio-Rad Laboratories Inc., Hercules, CA, USA) for at least 5 min at room temperature, membranes were left to react overnight at 4 °C with anti-Akt, anti-p-Akt (Ser473), anti-Caspase-3, anti-mTOR, anti-p-mTOR (Ser2448), anti-PARP, anti-p70S6K1, anti-p-p70S6K1 (Ser371), and anti-p-4E-BP1 (Thr37/46) (Cell Signaling Technology, Beverly, MA, USA) primary antibodies (1:1000 *v*/*v*) and anti-β-actin (Santa Cruz Biotechnology, Santa Cruz, CA, USA) antibody (1:2000 *v*/*v*). After rinsing three times with 1× PBS with 0.1% Tween 20 (PBST) for 10 min, membranes were subjected to incubation with horseradish peroxidase-conjugated anti-mouse or anti-rabbit IgG at a ratio of 1:3000 (*v*/*v*, Cell Signaling Technology) for 1 h at room temperature, and then rinsed three times with 1× PBST. The enhanced chemiluminescence solution from GE Healthcare Life Sciences (Little Chalfont, Buckinghamshire, UK) was used to visualize the bands, and the images were captured using the Fusion Solo 2M chemiluminescence imaging system from Vilber Lourmat (Marne-la-Vallée, France). Western blot bands were quantified using densitometry with ImageJ 1.52a software (US National Institutes of Health, Bethesda, MD, USA).

### 4.8. Animal Experimental Design

Twenty-four 3-week-old B6C3F1 female mice were purchased from Central Lab Animal Inc. (Seoul, Republic of Korea) and allowed to acclimatize for 1 week. Mice were randomly divided into four experimental groups (n = 6/group) as follows: control (Con), VCD (V), VCD + MFE low dose (100 mg/kg, V + ML), and VCD + MFE high dose (300 mg/kg, V + MH). Control mice received an intraperitoneal (i.p.) injection of sesame oil 5 times a week for 2 weeks. VCD and VCD + MFE-treated groups mice received an i.p. injection of VCD (160 mg/kg) 5 times weekly for 2 weeks. Water was used as a vehicle control for MFE. Starting 1 week before the first VCD administration, the vehicle or treatments were administered orally 5 times a week for 4 weeks. The day after the final doses, mice were anesthetized by i.p. injection with 125 mg/kg 2,2,2-tribromoethanol (Avertin^®^, Sigma-Aldrich), and blood samples were collected by cardiac puncture. Ovaries and uteri were excised for analysis and weighed. Animal care and use were conducted in accordance with institutional guidelines, and all animal experimental procedures were approved beforehand by the Institutional Animal Care and Use Committee of Dongguk University (IACUC-2020-005-3).

### 4.9. Histopathological Analysis

For histological examination, ovaries were removed, fixed in 4% paraformaldehyde overnight, and dehydrated in 70% ethanol, followed by paraffin embedding. Subsequently, sections of the tissues were prepared and underwent staining with hematoxylin and eosin (H&E). Stained sections were scanned with a whole-slide scanner (3DHISTECH Ltd., Budapest, Hungary) and evaluated using CaseViewer™ software (version 2.2, 3DHISTECH Ltd.).

### 4.10. Serum Biochemistry Analysis

Serum samples were obtained by centrifuging blood at 3000 rpm for 15 min at room temperature. Supernatants were collected and stored immediately at −80 °C in a deep freezer until required. To analyze hormone changes in serum, the levels of estradiol (E_2_) and anti-Müllerian hormone (AMH) were detected using an enzyme-linked immunosorbent assay (ELISA). E_2_ ELISA (Elabscience Biotechnology Inc., Houston, TX, USA) and mouse AMH ELISA (Cusabio, Wuhan, China) kits were used in according to the manufacturer’s instructions. Serum levels of aspartate aminotransferase (AST) and alanine aminotransferase (ALT) (key markers of liver function) were measured using the Asan set GOT Assay kit (Manual GOT) and the Asan set GPT Assay kit (Manual GPT) (both purchased from Asan Pharm., Seoul, Republic of Korea).

### 4.11. Statistical Analysis

The significance of differences between means was determined by one-way ANOVA and Tukey’s multiple comparisons using GraphPad Prism 5.0 software (GraphPad Software, Inc., San Diego, CA, USA). The results are presented as the means ± standard deviations (in vitro) or means ± standard errors (in vivo) of three or more independent experiments, and statistical significance was accepted for *p* values < 0.05.

## 5. Conclusions

Magnoliae Flos extract (MFE) demonstrated preventive effects in both cellular ovotoxicity and mouse models of premature ovarian insufficiency (POI) induced by VCD. MFE effectively protected ovarian cells from apoptosis and oxidative stress, while activating the Akt signaling pathway. In vivo, MFE administration increased ovarian health markers, suggesting its potential for preventing and managing conditions such as POI and diminished ovarian reserve. Despite the limitation of a small sample size, our study highlights the therapeutic potential of MFE in preserving ovarian function and warrants further investigations such as larger-scale studies and clinical trials to elucidate its mechanisms of action and its potential clinical applications in human reproductive health.

## Data Availability

The data supporting the findings of this study are available upon request from the corresponding author.

## Abbreviations

ALT, alanine aminotransferase; AMH, anti-Müllerian hormone; AST, aspartate aminotransferase; CHO, Chinese hamster ovary; DAPI, 4,6-Diamidino-2-phenylindole; DCFH2-DA, 2′,7′-dichlorofluorescein diacetate; DMEM, Dulbecco’s modified Eagle’s medium; E2, estradiol; H&E, hematoxylin and eosin; MF, *Magnoliae Flos*; MFE, MF extract; MH, MFE high dose; ML, MFE low dose; MTT, 3-(4,5-dimethylthiazol-2-yl)-2,5-phenyltetrazolium bromide; NOAEL, no observed adverse effect level; PARP, poly (ADP-ribose) polymerase; PBS, phosphate-buffered saline; PBST, PBS with 0.1% Tween 20; POI, premature ovarian insufficiency; ROS, reactive oxygen species; VCD, 4-vinylcyclohexene diepoxide.
